# Construct-Validating Humility: Perceptions of a Humble Doctor

**DOI:** 10.3389/fpsyg.2022.882622

**Published:** 2022-05-17

**Authors:** Sang-Yeon Kim, Erin Sahlstein Parcell

**Affiliations:** ^1^School of Media & Communication, Kwangwoon University, Seoul, South Korea; ^2^Department of Communication, University of Wisconsin-Milwaukee, Milwaukee, WI, United States

**Keywords:** humility, self-deprecation, source credibility, construct validation, impression management

## Abstract

Examining the nature of humility using self-report measures has been a challenging endeavor due to concerns of response biases and the common misconception that equates humility with self-deprecation. Alternatively, this study attempts to validate the construct of humility by assessing subjects’ (*N* = 553) responses to a speech written to represent the core elements of humility as opposed to self-deprecation or unconditional self-underrating. Data show that (a) humility comprises a latent construct subsuming accurate self-assessment, open-mindedness, and egalitarianism; and (b) humility outperforms self-deprecation in enhancing perceived sincerity, source credibility, and the intention to interact with the source. Results indicate, particularly for expert sources, that humility cues can promote approachability while maintaining perceived expertise.

## Introduction

Humility refers to a virtuous human characteristic ensuing balanced self-view, open-mindedness, and valuation of others (see Kachorek et al., 2004). Among scholars of personalcharacteristics, a consensus exists that there are at least two major challenges for studying humility. The primary challenge involves common misconceptions of humility. Quite frequently, the term humility is used to communicate concepts that differ qualitatively (e.g., self-deprecation, self-underrating; Emmons, 1984; Ben-Ze’ev, 1993; Morris et al., 2005). However, few have attempted to subject the claim to an empirical test. With the absence of relevant scientific evidence, precisely how humility differs from self-deprecating remains unknown. The second, more scholarly challenge concerns that humility may defy accurate assessment with self-reports (see Exline and Geyer, 2004; Davis et al., 2010). Because the measurement of humility uses items with embedded elements of social desirability, individuals with an overrated self-concept yet are self-conscious (e.g., narcissists) could misrepresent the self to appear humble. On the contrary, truly humble individuals may well describe themselves modestly, suppressing the true level of humility expressed. The end result could be that narcissists and humble individuals appear indistinguishable in self-reported measures.

As an alternative, Davis et al. (2010) proposed inviting couples to evaluate each other. The recommended remedy, however, is expected to create new problems, such as the inconvenience of having to recruit dyads or the non-independence within the paired responses. The conjecture that an individual’s humility level could fluctuate depending on the characteristics of the relationship, as well as the accompanied partner, may also weaken the potency of the alternative (see Tice et al., 1995). Similarly, Rowatt et al. (2006) have attempted to avoid the problem of adopting Implicit Association Tests (IATs) to assess humility by eliciting less controlled responses from the subjects. The measurements used, however, include direct questions asking the subjects to self-assess humility. Given the common misconception of humility, such responses may represent their self-judgments on a dimension (e.g., self-deprecation) other than humility.

More recently, Kruse et al. (2017) introduced a new measurement of state humility. The researchers shared the concern that humility is difficult to measure directly due to social desirability, and they attempted to address it by adopting items that explain the construct in lay terms instead of referring to humility or related concepts (e.g., modesty). Also, the researchers attempted to construct-validate humility by observing how it behaves when correlated with relevant, more established personality measures [e.g., HEXACO personality measures (Lee and Ashton, 2004), self-esteem (Rosenberg, 1965), narcissism (NPI, Raskin and Hall, 1979)].

Despite the fact that such efforts contribute to a better understanding of the true nature of humility, it should be noted that the State Humility Scale has two important limitations. First, the new 6-item measurement considers humility mainly as a lack of self-focus (e.g., *“I feel that, overall, I am no better or worse than the average person,” “I feel that I do not deserve more respect than other people”*). This operationalization runs counter to the literature that has consistently conceptualized humility as a multi-dimensional construct comprising three qualitatively different attributes (i.e., accurate self-assessment, open-mindedness, egalitarianism; see Morris et al., 2005; Tangney, 2005; Emmons, 2007). Such attempts may help attain a certain level of conceptual clarity but at the expense of examining the true, intricate nature of the construct as a whole. This issue also questions the validity of the construct validation results; the study demonstrates how “a part of humility” associates with other personality measures but *not* humility in itself.

Second, the measurement items seemingly tap into a construct conceptually similar to egalitarianism, one of the three components of humility. But a critical view reveals that the two differ in an important way. The items used in that particular study involve the self-assessment that one is just like everyone else and no better than others. Egalitarianism also connotes a sense of equity but in a much more positive and active tone. The humility literature has established that egalitarianism refers to the belief that one is born talented *and* so are all others, and people are only different in the kinds of talents that they possess (see Richards, 1992; Morgan, 2001). This notion thus allows judging one’s superiority to the next person in the kinds of talents highlighted at the moment. That is, in humility individuals are presumed to be equal in that they all are gifted in some sense yet differ in that their talents are unique-different in kind. Current items, albeit apparently intended by the authors for conceptual clarity, do *not* seem capable of fully communicating such conceptual intricacies of humility.

Importantly, the anticipated bias in self-assessment precludes construct validation of humility. To determine accurately the conceptual location of humility within the body of other related personal characteristics, and particularly to discern it clearly from similar yet theoretically distinct constructs (e.g., self-deprecation), minimizing the biases in the measurement of humility becomes imperative. Unveiling the nature of humility or characterizing the mentality of humble individuals becomes elusive with the known bias of self-report measures.

The present study offers an alternative to examine humility as initially theorized yet to avoid the measurement bias of self-report. Specifically, this study presents a transcript of a speech created to embody all major conceptual components of humility and subject it to the reader’s evaluation. A message evaluation approach improves upon the limitations of the existing self-report measures for at least two reasons. First, asking to evaluate someone, *not* the participants themselves, moves the locus of examination. The anticipated issue of social desirability can thus be avoided. Second, the alternative proposes measuring humility *indirectly* by tapping its sub-constructs better understood by lay individuals and in turn treating it as a social construct communicated within the interaction. As aforementioned, the misconception of humility seems prevalent. While scholars define humility as a distinct human virtue, it is commonly equated with self-deprecation in daily conversations. Humility, by definition, must entail depressed self-presentation, but the reverse is probabilistic because self-deprecators would underrate the self or “*play humble*” for impression management without being truly humble. This discrepancy between the scholarly and the lay definition of humility disallows measuring humility using direct questions. Data obtained by using items, such as *“how humble do you think you are?*,” would only tell us about participants’ attitudes toward self-deprecation and should add little to understanding the true nature of humility. Estimating the level of humility indirectly by assessing its components on which the theoretical definition accords closely with the lay conception should thus be a reasonable alternative.

The indirect measures also provide sufficient information to examine humility as a latent construct as recent structural equation modeling (SEM) techniques enable the examination of second-order models. Dillard and Shen (2005) have successfully adopted a similar approach to measure psychological reactance, which has been considered impossible to operationalize for decades, by treating it as a latent construct comprising the direct measurements of anger and negative thoughts simultaneously.

We anticipate the current study to present theoretical and practical implications mainly for scholars of interpersonal communication as the proposed methodology ensues a natural relocation of the research focus from personality to perception. That is, the major interest of this study is to document the evidence needed to determine the manner in which using versus *not* using humility cues creates perceptual differences in such dimensions as interpersonal attraction, source credibility, approachability, etc. in addition to relevant personality measures (e.g., self-esteem, narcissism, self-consciousness). Whether or not the proposed new measurement set can be immediately self-administered is of secondary interest at the moment and would require a separate investigation. Nonetheless, this should *not* overshadow the significance of the current research, especially for researchers of impression management and source credibility, to whom how humility cues are *communicated* would matter much more than determining if one is truly humble. Communicative aspects of humility have been examined in more recent studies, particularly in political leadership and persuasion. While the prior research document some evidence of persuasive impacts of humility cues, the results cannot be considered conclusive due to the difference in humility induction. For instance, D’Errico (2019) and D’Errico et al. (2022) induced humility by manipulating linguistic elements of a message (e.g., using non-directive instead of directive verbs, first-person plural over singular); D’Errico (2020) operationally defined humility based on participants’ responses instead of employing a controlled manipulation. This warrants a reexamination of humility by adopting an experiment that manipulates the actual contents of a message such that the true humility condition includes cues representing all of its conceptual components.

The present study attempts to (a) provide an alternative measurement of humility and (b) establish its construct validity particularly by demarcating it from self-deprecation or unconditional self-underrating. With an extensive literature review, this study conceptualizes humility as a latent construct subsuming accurate self-assessment, open-mindedness, and egalitarianism. The analysis follows adopting SEM to compare perceived humility to perceived self-deprecation on selected criterion measures (e.g., perceived sincerity, source credibility, interpersonal attraction, approachability). Instead of using self-report measures, the current study adopts perceptual responses to the self-introduction of a fictitious speaker, which was created to represent the three conceptual components of humility. The results should help scholars examine humility more accurately, the correct misconceptions about humility, and provide practical advice for sources inquiring about an efficient self-presentation strategy.

## Literature Review

### Defining Humility

Humility has been conceptualized similarly across branches of humanities and social sciences, including literature on leadership (Morris et al., 2005), clinical psychology (Tangney, 2005), positive psychology (Fullam, 2009), philosophy (Ben-Ze’ev, 1993), theology and spirituality (Emmons, 2007), community engagement (Harrell and Bond, 2006), impression management (Sedikides et al., 2007), counseling (Means et al., 1990), and culture (Choe et al., 2019).

Synthesizing the past research, three source characteristics have been consistently used to define humility: (a) accurate self-assessment, (b) open-mindedness, and (c) egalitarianism. Accurate self-assessment refers to an individual’s understanding and acknowledgment of their strengths and weaknesses (e.g., Morris et al., 2005) attempting neither to overestimate their worth nor underestimate their merits or achievements (Emmons, 1999). Accurate self-assessment is thus associated with a person having a ‘balanced’ self-view, which requires self-acceptance, acknowledgment of their superiority and merits, awareness of their importance, self-confidence, *and at the same time*, an understanding of, and the willingness to face, their imperfections and limitations (Emmons, 1999; Harrell and Bond, 2006). Subsuming this sense of balance in self-awareness, humility has been conceptualized as “a crest of human excellence between arrogance and lowliness” (Morris et al., 2005, p. 1331), a realistic appraisal of positive and negative characteristics (Sandage and Wiens, 2001), a prudential mean between unlimited self-denial and excessive self-orientation (Fullam, 2009), or a moderate self-view neither too positive nor too negative (Sedikides et al., 2007).

Open-mindedness denotes the mindset of remaining receptive to new ideas and paradigms (Tangney, 2005) or outgroup culture (Choe et al., 2019), and readiness to connect with, and learn from, others (Morris et al., 2005) especially those promoting contradictory beliefs and practices (Harrell and Bond, 2006) or possessing superior or different qualities and skills (Casey, 2001). This component of humility is a manifestation of the awareness that they are competent but can never be omniscient, and in this wide universe, there must exist individuals with superior qualities, who can better perform a task that they believe to possess expertise at or falsify their beliefs or opinions. Humility thus “opens a person to the possibility that [they do] not know the best, that [their] information is incomplete or [their] inferences faulty” (Richards, 1992, p. 198), enabling their willingness to acknowledge and face their limitations, mistakes, and imperfections (Fullam, 2009). With such a large perspective of the society, humble individuals hardly suffer from jealousy or feelings of suppression in face of superior others (Richards, 1992), experience embarrassment when having to learn from them (von Hildebrand, 1976), or deem others a competitor (Casey, 2001).

Egalitarianism involves the view that all humans are created equal, possessing equally valuable talents albeit of different sorts (e.g., Morgan, 2001). A humble person thus acknowledges their talents but also believes that all other souls are equally talented. Such individuals are predisposed to construe others as their equals and understand that none, including themselves, deserve special treatment (Richards, 1992). A renowned doctor, for instance, would be aware that they are talented in making accurate diagnoses with limited albeit particular information about their patients. Yet, the humble doctor might become humbled by a visiting technician repairing an appliance in minutes given this is not their expertise.

The discussion of humility, albeit abundant and ripe, still remains at the conceptual level, and few have attempted to construct-validate or operationalize humility by adopting scientific methods. Guided by the existing literature, the current investigation seeks to determine if humility could indeed be operationally defined as a latent construct comprising measures of accurate self-assessment, open-mindedness, and egalitarianism.

*RQ: Does humility comprise three sub-constructs, including accurate self-assessment, open-mindedness, and egalitarianism?*

### Construct-Validating of Humility

This study seeks to establish and validate a scale to measure the construct of humility. A classic method examines the extent to which the scale of the new construct correlates with the measures of other established constructs as predicted by theory (Campbell and Fiske, 1959). Items measuring the new construct should covary substantially with those of other constructs that are theoretically predicted to covary (i.e., convergent validity), while minimally correlating with those of constructs that are presumed theoretically independent (i.e., discriminant validity). This study examines the manner in which the measurements of humility behave when correlated with perceptions of sincerity, narcissism, self-consciousness, and source credibility. To demarcate humility from self-deprecation, perceived self-deprecation is also measured and subjected to the same set of analyses for comparison. In what follows, we define self-deprecation *in comparison to* humility and present predictions on the perceptual difference between the two concepts in levels of sincerity, narcissism, self-consciousness, and source credibility.

#### Self-Deprecation

Self-deprecation refers to one’s intentional discount of merits or disclosure of shortcomings, usually exercised by sources with superior qualities in an attempt to enhance interpersonal approachability (Blau, 1960). Recognizing that the source possesses excelling qualities, the audience would build barriers and avoid initiating social contacts with the source. Blau (1960) recommends that sources with superior qualities use self-deprecation to lower such barriers (Ross et al., 1977). Self-descriptions that are either “too good to be true” (i.e., self-enhancement) or “too bad to be true” (i.e., self-deprecation) deviate from normality, and this expectancy violation can produce unintended negative responses (Hareli and Weiner, 2000). In particular, “insincere,” “dishonest,” or “in need of more knowledge about self” formed the dominant impressions in the audience exposed to self-deprecators (Robinson et al., 1995).

Differentiating humility from self-deprecation can be particularly difficult noting the multi-dimensionality of humility. Based on the evidence from a multi-method study, Weidman et al. (2018) propose that humility takes two distinct forms: appreciative and self-abasing humility. The former is said to involve celebrating personal success as well as others’, promoting a sense of pride, whereas the latter concerns negative self-views, raising the tendency to focus on personal failures and avoid evaluations from others. Self-deprecation is conceptually similar to “self-abasing” humility in that it stresses one’s shortcomings. The existing definition of humility, on the other hand, encompasses both forms of Weidman et al.’s conceptualization as it requires a balanced self-evaluation; acknowledging the strengths *and* the weaknesses of oneself is at the very basis of humility. This notion indicates that self-deprecation constitutes a sub-dimension of humility, and therefore, distinguishing one from the other can be difficult especially when the source speaks of his/her limitations.

#### Sincerity

Past literature concur that a person who is self-deprecating or presents a *false* humility (see Driver, 1989; Sandage and Wiens, 2001), is engaging in an untruthful underrating of the self that is intended to manage their public image (Ben-Ze’ev, 1993). An expert source stated that they have little expertise, while *not* believing, it is exercising self-deprecation. In particular, perception of insincerity may arise in the message recipients who would believe that the biased self-presentation was executed intentionally (see Robinson et al., 1995).

In contrast, humility, as it requires possession of accurate or balanced self-assessment, involves a person having full awareness of their importance, strengths, and talents (Richards, 1992) while *not* intending to inflate such qualities in the eyes of others (Casey, 2001). Self-confidence, independence, or assertiveness are qualities conceptually closer to humility than self-skepticism, self-monitoring, or self-belittling for impression management or self-protection (Neuringer, 1991). Humble persons should be able to reveal what they can and cannot do openly and readily.

Humility thus precludes intentional self-denial or self-disparagement (Sandage and Wiens, 2001), denial of superior positions or accomplishments (Ben-Ze’ev, 1993), depressed opinion of the self (Emmons, 1999), self-abasement (Fullam, 2009), lowly demeanor (Neuringer, 1991), decreased valuation of the self (Means et al., 1990), or unduly underestimating the self, particularly for purpose of impression management (Driver, 1989). Humility does *not* “consist in handsome people trying to believe they are ugly, and clever people trying to believe they are fools…” (Buri, 1988, p. 93). A truly humble expert, for example, would express gratitude for a compliment about their expertise instead of repelling adulation (see Vera and Rodriguez-Lopez, 2004). Humble remarks may be perceived as less polite than the self-deprecatory response, but they may be viewed as more truthful or authentic than the latter.

The conceptual definition of accurate self-assessment, as a component of humility, predicts an increase in perceived sincerity because of the admission of strengths *and* weaknesses, which is conceptually equivalent to balanced self-presentation. The existing evidence for a positive impact of balanced self-presentation on the perception of sincerity provide the same prediction for humility (see Robinson et al., 1995). The first set of hypotheses derives from this line of thinking.

*H1a: Perceived humility and perceived sincerity correlate positively.*

*H1b: Perceived self-deprecation and perceived sincerity correlate negatively.*

#### Narcissism

Humility necessitates possession of an egalitarian worldview as mentioned above, which entails valuing others as much as oneself, assuming that all individuals in the universe possess unique talents or qualities to be used for society. A postulate arises that humble persons remain less self-centered than individuals who are *not*. The absence of narcissism cannot be automatically translated into the presence of egalitarianism because narcissism and egalitarianism are unlikely to be bipolar opposites. Nonetheless, noting that narcissists tend to be excessively self-oriented and obsessed with an inflated sense of self-importance (Emmons, 1984), humility, which comprises an egalitarian worldview, seems unlikely to manifest such qualities. Consistently, lack of narcissistic orientation (Vera and Rodriguez-Lopez, 2004) and low self-focus or self-loss (Peterson and Seligman, 2004) have been identified as one of the major qualities of humility. Morris et al. (2005) expect a negative correlation between humility and narcissism for the same reason.

Self-deprecation, in contrast, is likely to associate positively with narcissism noting that at least two necessary conditions must be satisfied for one to decide to self-deprecate. First, the source must be aware that they are superior to the counterpart(s) at least in the domain being discussed at the moment. That is, execution of self-deprecation necessitates possession and awareness of superior qualities, without which self-underrating is impossible. Second, the source should be aware that the audience acknowledges or will learn about the superiority or achievement (Schlenker, 1980).

The combination of the awareness that one possesses excellent qualities and the presumption that the superiority is honored by the audience may boost the source’s self-esteem, and in an extreme case, could push the source to a narcissistic end albeit temporarily. This conjecture seems reasonable noting that self-perception as *“I know I am talented, and they know it too”* is compatible with narcissism which is characterized by the belief that *“I am the center of attention.”* The postulate that self-deprecators can appear narcissistic becomes more probable assuming further that self-deprecation lacks egalitarianism, unlike humility. Individuals admitting qualification, but reluctant to believe that all other individuals are qualified, could hardly become *not* narcissistic particularly when cognizant of the respect from the audience. The discussion thus far leads to the second set of hypotheses.

*H2a: Perceived humility and perceived narcissism correlate negatively.*

*H2b: Perceived self-deprecation and perceived narcissism correlate positively.*

#### Self-Consciousness

Humility may differ from self-deprecation minimally in the manner in which they are enacted in social interaction as both qualities would ensue politeness or verbal decency. A polite self-presentation should thus obfuscate if it represents possession of humility or a deliberate attempt at impression management. Still, humility differs from self-deprecation in what motivates politeness. Humility provides an internally stable predisposition, possession of which can lead to a polite introduction of the self that is independent of social norms or situational demands. Self-deprecation, on the contrary, constitutes a self-presentation scheme adopted consciously to satisfy social requirements or to appear admirable at the moment. Therefore, under powerful social pressures, even an extreme narcissist can temporarily wear a look of politeness. Morris et al.’s (2005) example encapsulate this point:

Actors who thank their directors but truly believe that they were solely responsible for the film’s success are [self-deprecating] but not humble. This suggests that authentic humility leads to [self-deprecating] but that [self-deprecating] may not reflect true humility.[self-deprecation] is strongly subject to social rules and norms but may or may not reflect one’s true internal state. (p. 1332).

Ben-Ze’ev (1993) concurs that, for humble persons, polite behaviors stem from internal attributes, and thus the same polite behaviors can be enacted independent of social pressures. In contrast, “professionally humble” persons, or self-deprecators, are believed to act out politeness strategically to please the audience in that particular interaction. This notion justifies the conjecture that humble individuals remain less sensitive to social pressures than self-deprecators. Self-consciousness, more precisely public self-consciousness (Fenigstein et al., 1975), refers to the “awareness of the self as a social object that has an effect on others” (pp. 523). This conceptual definition well characterizes self-deprecators whose self-presentation is affected by the presence of others and driven by the motivation to impress them. The lack of association between humility and self-consciousness has been established repeatedly in prior research (see Ashton and Lee, 2005; Landrum, 2011). The third set of hypotheses is proposed as follows:

*H3a: Perceived humility and perceived self-consciousness are unrelated.*

*H3b: Perceived self-deprecation and perceived self-consciousness correlate positively.*

#### Source Credibility

Results from a recent study indicate that the use of self-deprecation cues outperforms using arrogance cues in improving perceived source credibility and likeability (Lapinski et al., 2007). Humility cues should help enhance source credibility and likeability even further because humility, as does balanced self-presentation, promises to lower the perception of insincerity, which constitutes a negative side effect of self-deprecation. Moreover, unlike self-deprecation, humility would preclude the concern that the source lacks knowledge about the self as accurate self-assessment constitutes the very foundation of humility. Thus, to the extent that sources perceived to self-present sincerely and accurately are to obtain a more favorable evaluation than sources perceived as insincere or with a biased self-image, a reasonable expectation that the same preference toward humble sources over self-deprecators exists as well.

At least in the United States, promoting critical thinking has been a primary educational goal pursued in almost every class across all grade levels and course contents. Thus, for the majority of the population with at least an intermediate level of education, critical thinking, particularly when applied in a self-reflective manner, should bear positive meanings as “educated” or “desirable” As this conjecture holds true, being open-minded or being egalitarian should induce positive source evaluation. The same conclusion is deducible from noting that their conceptual opposites, close-mindedness (e.g., *“No one is superior enough to falsify my position”*) or the belief that true talent is limited to a small segment of society, are compatible with arrogance, which has proven to lower source credibility and likeability in previous research (Lapinski et al., 2007). The current rationale leads to the fourth and the fifth hypothesis as below:

*H4: Perceived humility correlates with perceived source credibility more strongly and positively than perceived self-deprecation.*

*H5: Perceived humility correlates with perceived interpersonal attraction more strongly and positively than perceived self-deprecation.*

### Moderator: Outcome Involvement and Source’s Occupation

There exists a potential disjunction between source evaluation and the intention to interact with the source; as Blau (1960) posited, a source perceived as positive may *not* translate into an audience wishing to interact with the source. Therefore, a separate prediction of intention to interact is required.

The current rationale predicts that a humble expert will attract a target more strongly than would a self-deprecating expert. Individuals seek expert opinions and skills on task domains where they lack experience yet want to assure the quality of the outcome. A humble expert, presenting their merits freely and sincerely, is likely to be found more competent and hence more dependable than a self-deprecating expert, who would emphasize weaknesses over strengths. Provided that a humble expert is found more dependable than a self-deprecating expert, the former should attract more people (e.g., clients for a contractor, students for a teacher, patients for a doctor) than the latter.

The predicted difference between humility and self-deprecation in the extent of inducing intention to interact should increase as the source’s expertise generates a more substantive impact on the target. For example, expertise as a doctor can exert substantive yet immediate impacts as patients’ lives depend upon the accuracy of diagnosis and the quality of medical performance. Automotive technicians would also have substantive influence as skilled technicians are likely to isolate the cause of the problem accurately, precluding the recurrence of the same problem. Some expertise, on the other hand, have less substantive impacts. Expertise as a writer, for example, may provide moving experiences or times to think about values lost in our life, but is less likely to incur material benefits or costs. The evaluative contrast between humility and self-deprecation is likely more pronounced when tested with the former two than with the latter. Few would favor a self-deprecating doctor or an automotive technician as they may seem lacking in confidence or necessary skill sets. Writers, albeit with equivalent expertise, might make little perceptual difference whether they appeared humble or self-deprecating due to the insignificance of the influence.

Empirical findings well correspond to this notion. For example, influential Uber drivers running a YouTube channel have been identified to emphasize their unique know-how to earn popularity among the viewers pursuing a similar career (Chan, 2019); self-promotion tended to work to enhance the perceived competence of a female interviewee *without* compromising the social attraction and the hireability especially when the male perceivers were outcome-involved (Rudman, 1998); self-enhancement (e.g., clinical excellence, professional development, and training) surfaced as a common theme in the personal statements written by the applicants to United States internal medicine residency programs (Osman et al., 2015). The discussion thus far leads to the last prediction as below.

*H6: The positive impact of perceived humility on the intention to interact with the source is particularly stronger than that of perceived self-deprecation when the source has a more substantive impact on the receiver.*

## Materials and Methods

### Participants

Participants comprise 553 college students enrolled in introductory communication courses. Women constituted approximately 58% of the data. Approximately 95% of participants were 18 to 24 years old (*M* = 21.27, *SD* = 4.25). The majority of the participants were Caucasian (77%), followed by African American (9.3%), Hispanic (6%), Asian (6.6%), and other minorities (1.1%; Native Indians and Pacific Islanders). Among the participants, 95% reported that they were raised in the United States.

### Design and Procedure

To examine the predictions, this study employed a 2 (accurate, inaccurate self-presentation) × 2 (presence, absence of open-mindedness cues) × 2 (presence, absence of egalitarianism cues) × 2 (poet, doctor) between-subjects design. Participants were randomly assigned to one of the 16 conditions and read a transcript of a speech created to represent the designated combination of constructs. After reading the transcript, participants reported on dependent measures (i.e., behavioral intention to interact with the speaker, and perceived source credibility), manipulation check items, items for establishing discriminant validity (i.e., perceived sincerity, narcissism, and self-consciousness), measurements of control variables (i.e., perceived length of the speech), and demographics. In all 16 conditions, the person, K. T. Barrett, was fictitiously portrayed as a highly accomplished poet/doctor, who has published numerous poems/research articles widely accepted by the public/academia, with many students desiring to pursue the same life path as an influential poet/doctor.

The decision to use poet/doctor as the contrasting careers was to demonstrate that the predicted perceptual difference between humility and self-deprecation becomes much larger for a doctor as opposed to a poet. That is, patients are more outcome-dependent on the expertise of a doctor, hence expected to process the doctor’s self-presentation more effortfully. Consistently, past research shows that a more powerful induction is possible with subjects with an outcome involvement (see Johnson, 1994). Accuracy in self-assessment was manipulated by varying the extent to which the speaker admits/discounts the objective achievements provided in the introduction. The speaker with accurate self-assessment claims credit for the achievements as specified in the introduction (*“As introduced, I was a poet/doctor have been quite successful over the past years. I wrote many widely accepted poems/widely cited books and journal papers, and I believe that reflects my expertise and talents in poetry/dermatology”*), whereas the speaker with inaccurate self-assessment attributes the accomplishments to external causes (*“Your teacher has introduced that I am a successful poet/doctor but I am not quite sure. I wrote many poems/journal papers, but I believe it is because I was very lucky to have many inspirational/supportive colleagues around me*…”) and denies expertise (*“Though people would say that I am an expert, I’m still ignorant about poetry/dermatology and more often confused than I am clear about what poetry/dermatology is all about”*).

By definition, an accurate self-assessment should ensure a balanced presentation of self, in which both strengths and weaknesses must be mentioned. Accordingly, a qualification was added to the accurate self-assessment condition (“…*I must admit, however, that I definitely lack the ability to capture tiny nuances of life from everyday life/to make rapid diagnosis upon visual inspection*…*I still don’t have it because I have focused only on larger issues as life and death, god, reincarnation, etc./I still find myself being hesitant to make quick diagnoses because of my obsession with accuracy”*). This same limitation was also mentioned in the inaccuracy condition to assure equivalence in contents and hence commensurability among conditions. The addition of this limitation has strengthened the induction of inaccuracy in self-assessment noting that communicating demerits is conceptually consistent with self-deprecation.

The level of open-mindedness was varied by including or omitting a statement that acknowledges the potential existence of individuals with greater expertise, knowing better ways of structuring poems/producing diagnoses (“…*there exists thousands of poets/dermatologists in the United States alone*…*there must be many better ways to structure poems/to recognize skin lesions or a whole new perspective that may prove my way of structuring poems/formulating diagnoses is not as functional/accurate as I thought*…”) and the willingness to learn from the superior (*“I would be much glad to exchange ideas with such individuals so we can learn from each other/so I can improve my work”*).

Half of the subjects read the ending remarks reflecting the possession of egalitarianism or recognition of the ubiquity of talented individuals (*“I believe that every single individual in the world is born with equally valuable talents though they may be different in kind*…*I might be special in that I have talent as a poet/doctor, but at the same time, there is nothing special about me noting that everyone in the world is born in some sense gifted”*). These statements were omitted in the other half conditions. The full speech used to represent each of the 16 conditions can be obtained from the author.

### Measurements

The measurement of source credibility (McCroskey and Teven, 1999) used items identified through exploratory factor analysis (EFA). Factors were extracted using principal component analysis (PC) and Varimax rotated. As identified by McCroskey and Teven (1999), a three-factor solution summarized the data adequately, with each factor explaining a sufficient amount of the total variance. The factor structure maintained the original three dimensions of source credibility: expertise, trustworthiness, and goodwill. Only the items with factor loadings (L) surpassing 0.60 were adopted for analysis. The measurement of interpersonal attraction (McCroskey and McCain, 1974) underwent the same statistical procedure, which identified the conventional three-factor solution (i.e., social, task, and physical) as a sufficient representation of the data.

The measurement of open-mindedness comprised items from the Self-Righteousness Scale (SRS; Falbo and Belk, 1985). The SRS measures an individual’s level of self-confidence that opinions and beliefs are always correct, particularly when compared to those of others. Open-mindedness thus constitutes its opposite as it refers to when a person remains open to the possibility that their position can be falsified.

Egalitarianism items were adopted from the Belief in Equality Inventory (Gray et al., 1994), sincerity items from the Individualized Trust Scale (Wheeless and Grotz, 1977), narcissism items from NPI-16 (Ames et al., 2006), and self-consciousness items from the Self-Consciousness Scale (Fenigstein et al., 1975). All other measurements (e.g., accuracy in self-assessment, self-deprecation, perceived speech length, perceived amount of information made available about the source, perceived significance of the source’s career, evaluation of the speech, perceived conventional of the speech) were unavailable from past studies and thus created for the current study.

Intention to interact with the source was measured using 7-point Likert scales (1 = *strongly disagree*, 7 = *strongly agree*); measurements of source credibility employed 7-point Semantic Differential scales (e.g., 1 = *expert*, 7 = *inexpert*); all other variables were measured using 5-point Likert items (1 = *strongly disagree*, 5 = *strongly agree*). Reflected items were recoded such that a larger value indicates the greater of the target construct. Table 1 specifies the actual items excluding the ones removed to enhance the internal consistencies. Items measuring a common construct in an internally consistent manner were averaged to create composite indices.

**TABLE 1:** Items and item reliabilities.

Construct	α	Items
Intention to Interact *Poet*	0.89	*I would:* “be interested in reading Barrett’s poems.” “be interested in buying Barrett’s books.” “like Barrett’s poems.” “recommend Barrett’s poems to my friends.”
Intention to Interact *Doctor*	0.95	*I would:* “see Dr. Barrett if I had a skin problem.” “ask Dr. Barrett to examine me if I had a skin problem.” “be satisfied with Dr. Barrett’s treatment if I were his/her patient.” “recommend Dr. Barrett to my friends having skin problems.”
Source Credibility *(Expertise)*	0.91	*Barrett is:* “intelligent-unintelligent (R),” “untrained-trained,” “expert-inexpert (R),” “knowledgeable-*not* knowledgeable (R),” “informed-uninformed (R), “bright-stupid (R),” “experienced-inexperienced (R)”
Source Credibility *(Trustworthiness)*	0.78	*Barrett is:* “honest-dishonest (R),” “untrustworthy-trustworthy,” “phony-genuine,” “unethical-ethical”
Source Credibility *(Goodwill)*	0.90	*Barrett:* “cares about the audience-does not care about the audience. (R)” “is concerned with the audience-is not concerned with the audience. (R)” “is likable-not likable (R),” “is warm-is cold (R),” “is pleasant-is unpleasant (R)”
Interpersonal Attraction *(Social)*	0.79	“I think Barrett could be a friend of mine.” “I would like to have a friendly chat with Barrett.” “It would be difficult to meet and talk with Barrett. (R)” “Barrett just wouldn’t fit into my circle of friends. (R)” “We could never establish a personal friendship with each other. (R)” “Barrett would be pleasant to be with.”
Interpersonal Attraction *(Physical)*	0.83	“Barrett is somewhat ugly. (R)” “I don’t like the way Barrett looks. (R)” “Barrett is not very good looking. (R)”
Interpersonal Attraction *(Task)*	0.89	“You could count on Barrett getting the job done.” “I have confidence in Barrett’s ability to get the job done.” “If I wanted to get things done I could probably depend on Barrett.”
Accuracy in self-assessment	0.87	*Barrett:* “is well aware of his/her ability.” “has accurate perception of him/herself.” “knows his/her strengths and weaknesses.” “assessed his/her qualities accurately.” “accurately reflected the social evaluation of him/her.”
Open-mindedness	0.86	*Barrett would:* “enjoy having different points of view.” “be excited by the free exchange of ideas.”
Egalitarianism	0.75	*Barrett would believe that:* “the capacity for growth and improvement are equally great in everyone.” “there are people of unusual potential all around us.” “people have different talents that are equally valuable to society.”
Self-deprecation	0.94	*Barrett:* “underrated him/herself.” “discounted his/her achievement.” “underestimated his/her talents.” “undervalued his/her expertise.” “belittled him/herself.”
Self-consciousness	0.82	*Barrett would:* “be concerned about the way he/she presents him/herself.” “be self-conscious about the way he/she looks.” “usually care about making a good impression.” “usually be aware of his/her appearance.”
Sincerity	0.82	*Barrett is:* “sincere,” “candid,” “honest,” “trustworthy,” “unpretentious”
Narcissism	0.91	*Barrett would:* “believe he/she is a special person.” “like to be the center of attention.” “be apt to show off if he/she gets the chance.” “believe he/she is more capable than others.” “believe he/she is better than most others.” “look narcissistic to some people.”
Speech length	0.84	*Barrett’s self-presentation was:* “long,” “lengthy,” “wordy,” “short (R),” “brief (R)”
Amount of information	0.87	“Barrett provided enough information for me to assess his/her characteristics.” “The speech contained enough information about Barrett.” “I was able to make an informed evaluation of Barrett.” “Barrett disclosed him/herself enough for me to figure out his/her personalities.”
Career significance	0.91	*Barrett’s work as a poet/doctor:* “benefits the society in important ways.” “makes substantial contribution to our society.” “is important to our society.” “exerts considerable influence on people’s lives.” “affects people’s lives significantly.”
Speech Evaluation	0.89	*Barrett’s self-presentation was:* “powerful,” “inspiring,” “appropriate,” “effective”
Conventional	0.89	*The way that Barrett presented him/herself:* “was conventional.” “followed routines.” “reflects how most people would self-introduce in general.” “conforms to the social norm.” “was common.” “was normal.”

## Results

### Manipulation Check

A 2 (accurate, inaccurately underrated self-presentation) × 2 (presence, absence of open-mindedness cues) × 2 (presence, absence of egalitarianism cues) × 2 (poet, doctor) ANOVA was conducted to determine success of manipulations. Perception of accuracy in self-assessment was induced in the intended direction. Participants assigned in the accuracy condition (*M* = 3.75, *SD* = 0.66, *n* = 274) found the speaker more accurate in assessing the quality of the self than participants in the inaccuracy condition (*M* = 3.45, *SD* = 0.86, *n* = 279), *F*(1, 537) = 18.44, *p* < 0.01, η^2^ = 0.03. All other main impacts or higher order interactions were statistically non-significance at level α = 0.05. The induction of accurate/inaccurate self-assessment was more powerful when tested with perceived self-deprecating. Participants in the inaccuracy condition (*M* = 3.48, *SD* = 0.84, *n* = 279) rated the target as more self-deprecating than participants in the accuracy condition (*M* = 2.43, *SD* = 0.81, *n* = 274), *F*(1, 537) = 211.95, *p* < 0.01, η^2^ = 0.28. All other impacts were statistically non-significant at level α = 0.05.

Perception of open-mindedness was unaffected by the inclusion/exclusion of open-mindedness cues, *F*(1, 537) = 1.97, *ns*. Instead, varying levels of accuracy, *F*(1, 537) = 19.89, *p* < 0.01, η^2^ = 0.03, or varying levels of egalitarianism, *F*(1, 537) = 5.05, *p* < 0.05, η^2^ = 0.01, induced the perception of open-mindedness. One potential cause of the induction failure seems to be a mismatch between the measurement and the statements used for the speech. While the measurement involved the readiness to entertain *different* ideas, the speech was written to represent ‘open-mindedness’ included an acknowledgment of the existence of others with *superior* qualities who could prove the speaker incompetent. Results from a future analysis involving either the condition or the perception of open-mindedness should be interpreted cautiously.

Including/excluding the egalitarianism message produced perceptual difference in the intended direction. Participants found the speaker more egalitarian when exposed to the message that reflects possession of egalitarianism (*M* = 3.98, *SD* = 0.67, *n* = 275) than when *not* (*M* = 3.66, *SD* = 0.72, *n* = 276), *F*(1, 535) = 30.45, *p* < 0.01, η^2^ = 0.05. Perception of egalitarianism was also affected by varying levels of accuracy in self-assessment. The speaker with inaccurate self-assessment – that is, self-deprecation – (*M* = 3.91, *SD* = 0.68, *n* = 279) received a higher rating in egalitarianism than the speaker who presented the self accurately (*M* = 3.73, *SD* = 0.74, *n* = 272), *F*(1, 535) = 10.92, *p* < 0.01, η^2^ = 0.02. It should be noted, however, that the size of the impact for accuracy is much smaller.

The manipulation of career significance was successful. Participants rated that the doctor (*M* = 3.71, *SD* = 0.71, *n* = 259) can exert more substantive impacts than the poet (*M* = 3.28, *SD* = 0.73, *n* = 291), *F*(1, 548) = 46.51, *p* < 0.01, η^2^ = 0.08. All other perceptions remained unaffected by varying the source’s occupation. Table 2 specifies the descriptive statistics per each experiment condition, and Table 3 presents the main and the interaction effects of the experiment conditions for all the major outcomes of interest.

**TABLE 2:** Descriptives by message condition.

					BI		Expertise		Goodwill		Trust		Social_AT		Physical_AT		Task_AT		Accuracy	
Source	Accuracy	Open-Mind	Egalitarian	*n*	*M*	*SD*	*M*	*SD*	*M*	*SD*	*M*	*SD*	*M*	*SD*	*M*	*SD*	*M*	*SD*	*M*	*SD*
Poet	Deprecating	No	No	49	4.08	1.37	5.04	1.05	5.08	1.17	5.40	1.03	3.23	0.68	3.27	0.60	3.67	0.77	3.31	0.84
			Yes	30	4.62	0.99	5.20	0.74	5.36	0.85	5.58	0.88	3.45	0.60	3.26	0.61	3.40	0.69	3.61	0.70
		Yes	No	27	4.29	1.12	4.99	1.08	4.99	0.98	5.22	0.85	3.15	0.59	3.48	0.63	3.37	0.70	3.53	0.81
			Yes	42	4.18	1.24	5.05	1.02	5.30	1.25	5.45	1.21	3.26	0.76	3.01	0.44	3.45	0.77	3.49	0.79
	Accurate	No	No	35	4.14	1.19	5.42	0.99	4.73	1.38	5.21	1.08	3.00	0.66	3.18	0.72	3.63	0.68	3.75	0.63
			Yes	35	4.18	1.16	5.29	0.89	4.89	1.12	5.09	1.06	3.21	0.56	3.19	0.61	3.49	0.83	3.86	0.55
		Yes	No	29	4.34	1.06	5.14	0.95	4.57	1.18	5.03	0.92	3.06	0.76	3.05	0.35	3.45	0.59	3.66	0.86
			Yes	44	3.95	1.28	5.06	1.03	4.45	1.40	4.83	1.00	2.95	0.59	2.97	0.59	3.42	0.81	3.59	0.77
Doctor	Deprecating	No	No	37	3.87	1.63	4.92	1.11	4.96	1.16	5.42	1.19	3.06	0.70	3.21	0.61	3.33	0.86	3.44	0.83
			Yes	24	4.61	1.49	5.27	1.22	5.37	1.11	5.83	1.11	3.47	0.58	3.07	0.50	3.72	0.94	3.63	1.03
		Yes	No	32	3.63	1.41	4.65	1.36	4.68	1.45	5.14	1.03	3.31	0.64	3.28	0.62	2.92	1.05	3.40	0.91
			Yes	36	4.14	1.47	5.00	1.02	5.22	1.11	5.43	1.03	3.24	0.66	3.14	0.47	3.43	0.97	3.33	0.97
	Accurate	No	No	30	4.79	1.22	5.24	1.25	4.85	1.35	5.15	0.97	3.20	0.41	3.15	0.70	3.64	0.81	3.81	0.59
			Yes	35	5.35	1.14	5.79	0.92	5.22	1.14	5.43	1.08	3.28	0.63	3.30	0.48	3.75	0.89	3.81	0.54
		Yes	No	36	4.47	1.45	5.25	1.04	4.92	1.18	5.10	0.89	3.12	0.62	3.15	0.64	3.47	0.94	3.86	0.55
			Yes	29	4.56	1.44	5.41	1.13	4.43	1.17	5.05	1.04	2.98	0.61	3.02	0.53	3.80	0.87	3.69	0.72
Poet	Deprecating	No	No	49	3.37	0.90	3.72	0.84	3.75	0.76	3.23	0.80	3.70	0.62	2.51	0.78	2.73	0.80	3.00	0.93
			Yes	30	3.51	0.80	3.97	0.51	4.07	0.56	3.28	0.81	3.85	0.47	2.55	0.75	2.83	0.64	3.61	0.63
		Yes	No	27	3.26	0.73	3.61	0.90	3.73	0.70	2.96	0.74	3.61	0.60	2.84	0.78	2.76	0.78	3.24	0.94
			Yes	42	3.52	0.79	4.07	0.64	4.11	0.68	3.05	0.71	3.61	0.69	2.55	0.80	2.82	0.79	3.20	0.92
	Accurate	No	No	35	2.31	0.82	3.46	0.73	3.34	0.74	3.48	0.81	3.49	0.62	3.42	0.80	2.92	0.65	3.04	0.96
			Yes	35	2.45	0.73	3.67	0.90	4.01	0.55	3.44	0.64	3.57	0.61	3.13	0.90	3.10	0.68	3.26	0.82
		Yes	No	29	2.26	0.67	3.31	0.99	3.44	0.71	3.34	0.77	3.39	0.71	3.52	0.60	2.91	0.68	3.00	0.90
			Yes	44	2.30	0.92	3.61	0.96	3.82	0.77	3.11	0.65	3.29	0.74	3.30	0.99	3.03	0.73	3.09	0.89
Doctor	Deprecating	No	No	37	3.46	0.92	3.66	0.65	3.73	0.55	3.17	0.83	3.71	0.70	2.30	0.66	2.72	0.81	2.81	0.95
			Yes	24	3.41	0.86	3.67	0.89	4.17	0.56	3.34	0.60	3.86	0.68	2.41	0.80	2.62	0.80	3.39	0.75
		Yes	No	32	3.70	0.74	3.91	0.79	3.84	0.69	3.22	0.78	3.63	0.58	2.42	0.74	2.48	0.83	2.84	0.81
			Yes	36	3.56	0.88	4.00	0.77	3.94	0.70	3.28	0.70	3.76	0.67	2.60	0.79	2.95	0.75	3.09	0.85
	Accurate	No	No	30	2.57	0.90	3.34	0.89	3.66	0.87	3.54	0.74	3.56	0.64	3.31	0.56	3.20	0.68	3.06	0.68
			Yes	35	2.49	0.74	3.46	0.90	3.97	0.60	3.55	0.63	3.69	0.56	3.15	0.79	3.40	0.56	3.30	0.76
		Yes	No	36	2.68	0.86	3.69	0.73	3.73	0.65	3.34	0.76	3.52	0.68	3.09	0.70	3.06	0.81	2.91	0.86
			Yes	29	2.42	0.77	3.53	0.94	3.78	0.82	3.54	0.63	3.55	0.54	3.48	0.74	3.20	0.66	2.99	0.91

*BI = Behavioral intention to interact with the source. AT = Attraction. Open = Open Mindedness. Speech = Speech evaluation.*

**TABLE 3:** ANCOVA.

Source	Effect	HU	BI	EX	GW	TR	SO	PH	TS	AC	DP	OP	EG	CO	SI	NR	CV	SP
Doctor	A	1.20	6.45**	1.66	0.05	0.91	1.11	0.24	2.28	3.75*	17.78***	3.31	0.22	5.57*	0.84	31.60***	6.90**	1.58
	O	2.32	0.72	> 0.01	< 0.01	0.09	0.50	< 0.01	0.82	0.04	0.23	3.67*	0.21	2.08	0.12	1.66	0.64	0.71
	E	3.96*	2.81	3.67*	1.37	1.15	0.15	1.16	0.08	< 0.01	0.30	0.57	3.38	0.11	0.46	0.61	0.98	0.97
	A × O	0.25	0.01	0.39	0.52	0.33	2.73	0.09	0.39	0.09	0.21	0.29	0.02	1.47	0.01	1.89	0.13	0.43
	A × E	< 0.01	0.07	0.22	0.01	0.10	2.32	1.96	0.89	0.48	0.05	0.27	0.22	0.72	0.03	0.89	1.11	1.42
	O × E	2.13	1.02	0.97	3.76*	0.80	0.81	1.90	0.66	0.33	0.23	1.16	1.22	1.08	0.10	4.26*	0.02	0.17
	A × O × E	0.19	0.17	0.55	2.98	0.28	0.67	0.81	0.06	0.05	0.01	1.10	< 0.01	1.08	0.05	1.80	2.43	0.18
Poet	A	5.35*	< 0.01	2.64	2.15	1.04	2.66	0.44	0.26	6.19**	33.12***	2.32	7.78**	2.05	3.12	24.32***	1.24	0.04
	O	0.01	0.24	1.88	0.38	0.74	0.08	0.80	1.47	0.47	0.03	0.57	0.20	0.71	0.65	0.15	0.04	0.24
	E	10.27**	0.01	0.41	0.30	0.27	1.66	< 0.01	0.71	0.34	0.47	1.19	16.35***	0.06	0.25	2.23	0.98	1.22
	A × O	0.07	< 0.01	0.45	0.01	< 0.01	0.37	3.08	0.31	1.37	0.03	0.01	0.27	0.26	< 0.01	0.58	0.03	0.53
	A × E	1.20	0.75	0.42	0.04	0.44	< 0.01	0.04	0.57	0.16	0.04	< 0.01	2.89	0.20	< 0.01	0.78	0.37	0.48
	O × E	0.87	1.11	0.12	0.41	0.14	1.89	0.13	0.32	0.33	0.18	0.19	1.37	0.42	0.53	0.10	0.04	0.18
	A × O × E	1.47	< 0.01	0.01	0.45	0.20	0.61	2.42	0.81	0.10	0.19	0.17	1.59	0.67	0.11	0.82	0.04	0.43

A = Accuracy; O = Open-mindedness; E = Egalitarianism; HU = Humility estimated; BI = Behavioral intention to interact; EX = Expertise; GW = Goodwill; TR = Trustworthiness; SO = Social attraction; PH = Physical attraction; TS = Task attraction; AC = Accuracy perceived; DP = Deprecation perceived; OP = Open-mindedness perceived; EG = Egalitarianism perceived; CO = Self-consciousness perceived; SI = Sincerity perceived; NR = Narcissism perceived; CV = Conventional; SP = Speech evaluation. Statistics represent F-values. df_1_ = 1 for all the analyses; df_2_ = 247 and 280 for the ‘doctor’ and the ‘poet’ condition, respectively. Perceived amount of information about the source entered the model as a covariate. * p < 0.05, ** p < 0.01, *** p < 0.001.

### Hypothesis Testing

#### Construct-Validating Humility

RQ asked if humility is a second-order construct subsuming accurate self-assessment, open-mindedness, and egalitarianism. Examining RQ requires to first seeing if the three measurements would hold their own unique factors (EFA; Exploratory Factor Analysis) and then submitting the identified factor structure to confirmatory factor analysis (CFA) to determine the model-data fit. Accordingly, the dataset was randomly split into two (*n* = 269 per each), one to be used for EFA and the other for CFA. An EFA was first conducted including all the 10 items measuring accuracy in self-perception, egalitarianism, and open-mindedness. Analysis found the 3-factor model a sufficient summary of the data as predicted, χ^2^(42, *N* = 269) = 137.92, *p* < 0.001 (see Table 4 for full results). The scree plot confirmed the results, showing a diminishing return beginning at the inclusion of the 4*^th^* component.

**TABLE 4:** Exploratory factor analysis.

	F1	F2	F3
ACCU_1	0.656	< 0.100	< 0.100
ACCU_2	0.817	< 0.100	0.150
ACCU_3	0.626	0.239	0.170
ACCU_4	0.808	0.133	0.119
ACCU_5	0.707	0.105	0.112
EGAL_1	0.295	0.573	0.358
EGAL_2	< 0.100	0.675	0.174
EGAL_3	0.173	0.769	0.214
OPEN_1	0.161	0.211	0.896
OPEN_2	0.169	0.382	0.739

*ACCU = Accuracy items; EGAL = Egalitarianism items; OPEN = Open-mindedness items. Factors were extracted using Maximum Likelihood estimation and Varimax-rotated. OPEN_1, OPEN_4, and EGAL_2 were loaded strongly to none of the factors and thus removed from further analysis. The scree plot demonstrates a sharp decline in the diminishing return at the inclusion of the 4^th^ component, indicating the 3-factor solution is a sufficient summary of the data, χ^2^(42, N = 269) = 137.92, p < 0.001.*

A CFA followed with the remaining half of the data. The model included humility as a latent construct subsuming accuracy in self-perception, open-mindedness, and egalitarianism as established in the preceding EFA. The model-data fit was acceptable to the conventional criteria with χ^2^(32, *N* = 269) = 76.07, *p* < 0.001, CFI = 0.964, RMSEA = 0.072, RMSEA CI_90%_ = [0.051,0.092], SRMR = 0.046.

**FIGURE 1:**
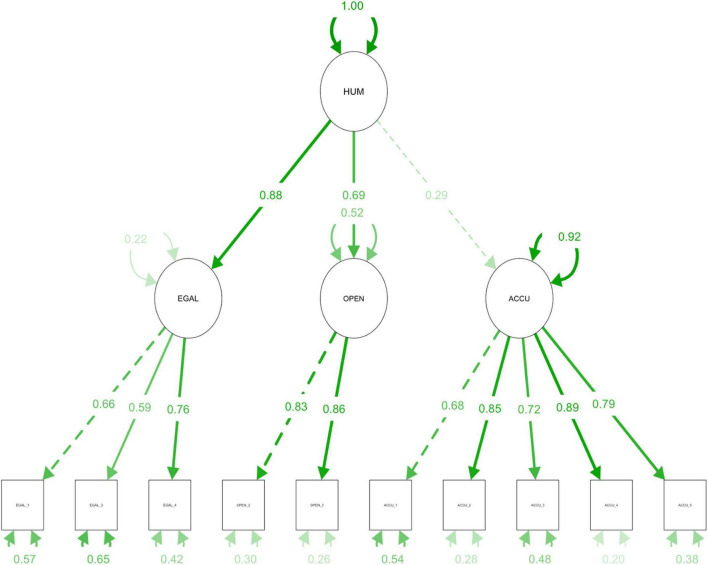
Factor structure of humility as a latent construct. HUM = Humility; EGAL = Egalitarianism; OPEN = Open-mindedness; ACCU = Accuracy. χ^2^(32, *N* = 269) = 76.07, *p* < 0.001, CFI = 0.964, RMSEA = 0.072, RMSEA CI_90%_ = [0.051,0.092], SRMR = 0.046.

It should be noted, however, that accuracy in self-perception (*L* = 0.29) tends to contribute much less to tapping into the latent construct compared to either open-mindedness (*L* = 0.69) or egalitarianism (*L* = 0.88). To adjust for this imbalance, the factor scores, which will constitute the predicted values of humility, were estimated using Bartlett’s approach, a refined method of calculating factor scores in an unbiased way (see DiStefano et al., 2009). Figure 1 illustrates the conceptual structure of humility.

To examine H1, H2, and H3, a correlation matrix was established involving the factor scores of humility and the measurements of self-deprecation, perceived sincerity, perceived narcissism, and perceived self-consciousness of the source. Table 5 specifies the results.

**TABLE 5:** Full correlation matrix.

	Humility	1	2	3	4	5	6	7	8	9	10	11	12	13	14	15
Intent 1	0.33** 0.25**															
Expertise 2	0.39** 0.29**	0.41** 0.65**														
Goodwill 3	0.55** 0.50**	0.39** 0.34**	0.60** 0.55**													
Trust 4	0.51** 0.52**	0.39** 0.36**	0.61** 0.52**	0.71** 0.67**												
Social_AT 5	0.48** 0.40**	0.52** 0.34**	0.38** 0.36**	0.64** 0.63**	0.51** 0.54**											
Physical_AT 6	0.03 0.18**	0.03 0.13*	0.10 0.16**	0.19** 0.23**	0.20** 0.27**	0.22** 0.36**										
Task_AT 7	0.36** 0.28**	0.34** 0.62**	0.44** 0.66**	0.35** 0.44**	0.41** 0.47**	0.37** 0.37**	−0.02 0.17*									
Accurate 8	0.47** 0.42**	0.35** 0.40**	0.43** 0.47**	0.38** 0.36**	0.36** 0.36**	0.35** 0.33**	0.02 *0.10*	0.34** 0.44**								
Deprecating 9	0.12 0.07	0.02 -0.32**	-0.25** -0.36**	0.09 -*0.03*	0.14* -*0.01*	0.12* -*0.01*	0.05 -*0.02*	-0.09 -0.32**	-0.33** -0.36**							
Open mind 10	0.82*** 0.78***	0.30** 0.11	0.38** 0.16*	0.56** 0.41**	0.49** 0.42**	0.51** 0.37**	0.05 0.20**	0.30** 0.14*	0.31** 0.22**	0.11 0.22**						
Egalitarian 11	0.90*** 0.89***	0.27** 0.24**	0.31** 0.27**	0.42** 0.41**	0.42** 0.44**	0.35** 0.30**	0.01 *0.10*	0.33** 0.25**	0.32** 0.30**	0.13* 0.06	0.56** 0.48**					
Conscious 12	0.17** 0.23**	0.16** 0.37**	0.27** 0.37**	0.14* 0.28**	0.11 0.24**	0.11 0.22**	-0.02 *0.08*	0.25** 0.41**	0.22** 0.26**	-0.09 -0.28**	0.25** 0.09	0.06 0.22**				
Sincere 13	0.55** 0.60**	0.41** 0.26**	0.42** 0.38**	0.56** 0.63**	0.66** 0.71**	0.47** 0.58**	0.12* 0.28**	0.49** 0.47**	0.43** 0.40**	0.14* 0.05	0.44** 0.49**	0.49** 0.51**	0.09 0.21**			
Narcissism 14	-0.36** -0.24**	-0.06 0.25**	-0.09 0.24**	-0.43** -0.15*	-0.40** -0.23**	-0.35** -0.14**	-0.17** -*0.04*	-0.19** 0.17**	-0.01 0.27**	-0.44** -0.51**	-0.34** -0.35**	-0.34** -0.20**	0.17** 0.22**	-0.37** -0.25**		
Speech Eval 15	0.47** 0.30**	0.50** 0.43**	0.39** 0.48**	0.47** 0.54**	0.39** 0.38**	0.50** 0.50**	0.06 0.21**	0.34** 0.48**	0.50** 0.42**	0.01 -0.24**	0.38** 0.23**	0.37** 0.22**	0.10** 0.40**	0.53** 0.44**	-0.19** 0.08	
Convention 16	0.17** 0.15**	0.27** 0.33**	0.17** 0.29**	0.17** 0.25**	-0.02 *0.08*	0.11 0.26**	-0.18* 0.09	0.11 0.28**	0.33** 0.32**	-0.08 -0.27**	0.11 0.14**	0.12* 0.08	0.33** 0.31**	0.09 0.16**	0.21** 0.32**	0.36** 0.48**

Estimates appear in regular font for the ‘poet (n = 292)’ and in italics for the ‘doctor (n = 262)’ condition. AT = Attraction. * p < 0.05, ** p < 0.01, *** p < 0.001.

H1 was mainly consistent with the data. Across conditions, humility correlated positively with perceived sincerity substantially with *r*_poet_ = 0.55 and *r*_doctor_ = 0.60. The perception of self-deprecating also had a positive association with perceived sincerity, *r*_poet_ = 0.14 and *r*_doctor_ = 0.05, but the strength of their associations remained trivial with η^2^ < 0.01. In sum, the current data point to a firm positive correlation between humility and perceived sincerity, and the likelihood of a null relationship between perceived self-deprecating and perceived sincerity.

H2 stated that perception of narcissism correlates negatively with humility and positively with perceived self-deprecating. Humility, as predicted, was inversely related with perceived narcissism, *r*_poet_ = −0.36 and *r*_*doctor*_ = −0.24, but so was self-deprecating; *r*_poet_ = −0.44 and *r*_doctor_ = −0.51. The distance between the two effects fell within sampling error for the poet, *z* = 1.15, *p* = 0.13. Analysis indicated, however, that the size of the impact for self-deprecation surpasses that for humility in a statistically significant manner when the source was a doctor, *z* = 3.62, *p* < 0.001. That is, a source either perceived as humble or self-deprecating is unlikely to be viewed as narcissistic, yet self-deprecating outperforms humility in preventing the source from being perceived as narcissistic particularly when the source is perceived to have expertise that might affect the recipients’ well-being.

The current data reversed H3’s prediction; self-consciousness correlated positively with humility, *r*_poet_ = 0.17 and *r*_doctor_ = 0.23, and negatively with self-deprecation, *r*_poet_ = −0.09 and *r*_doctor_ = −0.28. In particular, the two impacts differed significantly for the doctor, *z* = 5.94, *p* < 0.001. The results will be discussed in detail later in the manuscript.

#### Source Credibility, Interpersonal Attraction, and the Intention to Interact

The investigation continued to determine whether using humility cues or using self-deprecation cues enhances source credibility (H4) and interpersonal attraction (H5) more strongly. As mentioned above, the induction failure with open-mindedness unwarranted examining conditions, including open-mindedness cues. Accordingly, the subsequent analyses narrowed the focus to comparing quasi-humility (i.e., accurate self-assessment plus egalitarianism) to self-deprecation (i.e., inaccurate self-assessment) for source credibility and interpersonal attraction. Adding the source’s occupation as a moderator, the final model specified a 2 (quasi-humility, self-deprecating) × 2 (poet, doctor) between-subjects model.

Due to the additive nature of humility induction, the humility message became longer than the self-deprecation message, and it was presumable that perceived message length could confound the results. Accordingly, the perceived length of the message and the perceived amount of information provided by the source were entered into the model as covariates.

To examine H4, a MANCOVA was conducted on perceived expertise, trustworthiness, and goodwill simultaneously. Results from the omnibus test indicated a weak but significant main impact for message (quasi-humility, self-deprecation), Wilks’ λ = 0.88, *F*(3, 148) = 7, *p* < 0.01. A subsequent univariate analysis revealed that this statistical significance was driven by the message’s impact on perceived expertise, *F*(1, 150) = 11.68, *p* < 0.01, η^2^_*partial*_ = 0.07; across occupations, the quasi-humble source (*M* = 5.54, *SD* = 0.93, *n* = 70) was perceived to have a greater expertise than the self-deprecatory (*M* = 4.99, *SD* = 1.07, *n* = 86). The analysis found all other effects statistically non-significant at level α = 0.05, warranting no further analyses. The current finding indicates that quasi-humility enhances perceived expertise more powerfully than self-deprecating, while the two self-presentation tactics differ little in affecting perceptions of trustworthiness or goodwill. Data could thus be said partially consistent with H4.

The measurements of interpersonal attraction (i.e., social, physical, task) entered the same model in place of the measures of source credibility to test H5. All other features of the model remained the same. Results from the multivariate omnibus test demonstrated a significant message by job interaction, Wilks’ λ = 0.93, *F*(3, 151) = 3.59, *p* = 0.01. A univariate analysis revealed that this effect originates from differing perceptions of task attractiveness across conditions, *F*(1, 153) = 10.83, *p* = 0.001, η^2^_*partial*_ = 0.06. Specifically, Doctor Barrett was perceived more task-attractive when using humility cues (*M* = 3.81, *SD* = 0.87, *n* = 29) than when employing self-deprecation tactics (*M* = 3.33, *SD* = 0.86, *n* = 37). Using humility (*M* = 3.42, *SD* = 0.81, *n* = 44) or self-deprecation cues (*M* = 3.67, *SD* = 0.77, *n* = 49) produced little perceptual contrast when the source was a poet. A simple effect test, however, indicated that the difference in perceived task attractiveness within the doctor condition falls within sampling error, *F*(1, 155) = 2.14, *p* = 0.15, most likely due to the small sample sizes. As above, the current data were partially consistent with H5.

To examine H6, the same model predicted the intention to approach the source. Results from ANCOVA indicated a significant message by job interaction, *F*(1, 150) = 9.80, *p* < 0.01, η^2^_*partial*_ = 0.06. A subsequent contrast coefficient test (poet × self-deprecating, poet × humility, doctor × self-deprecating = -1; doctor × humility = + 3) confirms the prediction-data correspondence for H6, *t*(152) = 5.04, *p* < 0.01. Further analysis indicated a significant simple impact for message within the doctor condition, *F*(1, 152) = 21.23, *p* < 0.01, η^2^ = 0.14; participants were more willing to consult the doctor portrayed to be quasi-humble (*M* = 5.35, *SD* = 1.14, *n* = 35) rather than self-deprecating (*M* = 3.87, *SD* = 1.63, *n* = 37). The level of intention to interact remained almost invariant when the source was a poet, *F*(1, 152) = 0.32, *ns*; the quasi-humble poet (*M* = 4.18, *SD* = 1.16, *n* = 35) was found no more approachable than the self-deprecating poet (*M* = 4.08, *SD* = 1.37, *n* = 49). The disordinal interaction unwarranted further examination of main impacts.

**FIGURE 2:**
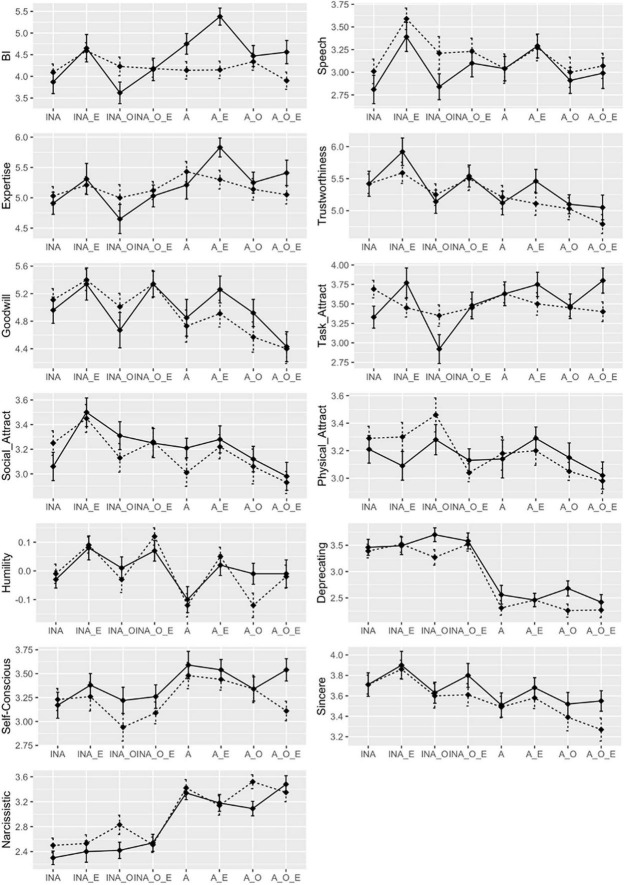
Message by source interaction on the major outcome variables. —- Poet — Doctor. BI = Behavioral intention to interact with the source; INA = Inaccurate self-perception or self-deprecation; INA_E = Self-deprecation + Egalitarianism; INA_O = Self-deprecation + Open-mindedness; INA_O_E = Self-deprecation + Open-mindedness + Egalitarianism; A = Accurate self-perception; A_E = Accurate self-perception + Egalitarianism; A_O = Accurate self-perception + Open-mindedness; A_O_E = Accurate self-perception + Open-mindedness + Egalitarianism. Error bars indicate CI_68%_.

Figure 2 illustrates how the perception of the source changes across the eight individual message conditions. The overall pattern of the data corresponds to the current results. Particularly for the doctor, task-related perceptions (i.e., intention to interact, expertise, task-attraction), (a) remain the lowest when the speaker self-deprecated (i.e., inaccurate self-assessment) *and* admitted the potential existence of more competent practitioners (i.e., open-mindedness), (b) turn positive when the self-assessment becomes accurate or balanced, (c) culminate when the remarks communicating an egalitarian worldview were added (i.e., quasi-humility), and (d) then decline with the further addition of open-mindedness cues (i.e., humility). A weak, reverse pattern was pronounced for the perception of social attributes of the source (e.g., trustworthiness, social attraction).

Also evident from Figure 2 is the accurate self-assessment functioning as a ‘switcher’ of perceived self-deprecation and narcissism; the former sharply declines and the latter uprises when the self-assessment turns from inaccurate (i.e., underrated self-presentation) to accurate (i.e., balanced self-presentation). Manipulating the other two components of humility had little impact on either perception. All other perceptions tended to fluctuate narrowly across conditions, precluding a coherent interpretation.

Combining the results from testing H4, H5, and H6, it is presumable that quasi-humility cues (i.e., accurate self-assessment + egalitarianism) help to enhance the intention to interact with the expert source by raising perceived superiority (i.e., task attractiveness, expertise), without rendering the source socially unattractive compared to a self-deprecating one. On the contrary, self-deprecating seems to only damage the source’s perceived expertise and approachability, providing little aid to boost the social dimensions of source credibility and attractiveness.

#### Reexamining Hypotheses With Perception Variables

The current findings cannot be considered conclusive noting the reduction in the conceptual breadth of humility; conditions including open-mindedness were excluded due to induction failure. Nevertheless, to the extent that it is the perceptual outcome from reading the message, *not* the message itself, that affects the judgments of the source, the impact of humility must be still testable indirectly by using *perceived humility* (see O’Keefe, 2002).

This rationale warranted revisiting the correlation matrix (see Table 5). Perception of humility produced ample positive impacts on all three components of source credibility (*r*_poet_ = 0.39 and *r*_doctor_ = 0.29 for expertise; *r*_poet_ = 0.55 and *r*_doctor_ = 0.50 for goodwill; *r*_poet_ = 0.51 and *r*_doctor_ = 0.52 for trustworthiness) and the intention to interact (*r*_poet_ = 0.23 and *r*_doctor_ = 0.25) while self-deprecation had little effects for personality components (*r*_poet_ = 0.09 and *r*_doctor_ = −0.03 for goodwill; *r*_poet_ = 0.14 and *r*_doctor_ = −0.01 for trustworthiness) of source credibility, and deleting impacts for perceived expertise (*r*_poet_ = −0.25 and *r*_doctor_ = −0.36) and the intention to interact particularly when the source was a doctor; *r*_poet_ = 0.02 and *r*_doctor_ = −0.32. Similar results continued when humility was correlated with the dimensions of interpersonal attraction. In particular, humility had a strong, positive impact on social attraction (*r*_poet_ = 0.48 and *r*_doctor_ = 0.40) and a moderate effect for task attraction (*r*_poet_ = 0.36 and *r*_doctor_ = 0.28). When the source was a doctor, perceived humility also predicted an elevated physical attraction (*r*_doctor_ = 0.18) albeit weakly. Perceived self-deprecation had a small, positive effect for social attraction when the source was a poet (*r*_poet_ = 0.12) but produced little impact when the source was a doctor (*r*_doctor_ = −0.01). The impact of perceived self-deprecation was minimal for physical attraction (*r*_poet_ = 0.05 and *r*_doctor_ = −0.02) and the task attraction for the poet (*r*_poet_ = −0.09) but substantially lowered the task attraction for the doctor (*r*_doctor_ = −0.32).

Assuming that perceived source credibility and perceived interpersonal attraction predict the willingness to interact with the source, it is opined that humility enhances the intention to interact *via* enhanced source credibility and interpersonal attraction while self-deprecation dampens the intention to interact by lowering perceived expertise and task attraction *without* improving perceived sociability. The data from the “doctor” participants especially provide a stronger support for this conjecture. For the doctor, perceived humility substantially outperformed perceived self-deprecation in raising the intention to interact (*r*_*hum*_ = 0.25, *r*_*dep*_ = −0.32, *z* = 6.68, *p* < 0.001), perceived expertise (*r*_*hum*_ = 0.29, *r*_*dep*_ = −0.36, *z* = 7.69, *p* < 0.001), goodwill (*r*_*hum*_ = 0.5, *r*_*dep*_ = −0.03, *z* = 6.59, *p* < 0.001), trustworthiness (*r*_*hum*_ = 0.52, *r*_*dep*_ = −0.01, *z* = 6.67, *p* < 0.001), social attraction (*r*_*hum*_ = 0.40, *r*_*dep*_ = −0.01, *z* = 4.94, *p* < 0.01), physical attraction (*r*_*hum*_ = 0.18, *r*_*dep*_ = −0.02, *z* = 2.3, *p* = 0.01), and task attraction (*r*_*hum*_ = 0.28, *r*_*dep*_ = −0.32, *z* = 7.05, *p* < 0.001).^1^ These findings are comprehensible noting that patients would want to see doctors appearing to be competent. Importantly, a competent yet sociable doctor seems to be the one that uses humility cues rather than relying on self-deprecating tactics.

### Supplementary Analysis

In an attempt to explore the rest of the data, the three main effects (i.e., accurate vs. inaccurate self-perception, presence vs. absence of open-mindedness cues, presence vs. absence of egalitarianism cues) and all of their possible combinatory impacts were established on the major dependent measures. Noting the additive nature of the experimental design, the perceived amount of information about the source was used as a covariate. Table 3 specifies the full results, including all higher-order interaction terms. Most of them, however, were trivial in size and deemed hard to replicate and thus subjected to no further discussion.

Three noteworthy, holistically coherent observations surfaced around the accuracy induction. First, accuracy induction exerted a far greater impact on the intention to interact when the source was a doctor [*F*(1, 247) = 6.45, *p* < 0.01, η^2^ = 0.03] than when the source was a poet [*F*(1, 280) < 0.01, *ns*], and examining the descriptive statistics, participants were more inclined to interact with the doctor with an accurate self-perception (*M* = 4.8, *SD* = 1.35, *n* = 130) than the one who would self-deprecate (*M* = 4.02, *SD* = 1.53, *n* = 129).

Second, the impact of accuracy induction for perceived self-deprecation was smaller for Doctor Barrett [*F*(1, 247) = 17.78, *p* < 0.001, η^2^ = 0.07; *M* = 2.55, *SD* = 0.82, *n* = 130 for the accuracy, and *M* = 3.54, *SD* = 0.84, *n* = 129 for the inaccuracy condition] than for Barrett the poet [*F*(1, 280) = 33.12, *p* < 0.001, η^2^ = 0.11; *M* = 2.33, *SD* = 0.8, *n* = 143 for the accuracy, and *M* = 3.42, *SD* = 0.82, *n* = 148 for the inaccuracy condition]. While the distance between the two effects fell within sampling error after *z*-transformation (*z* = -0.84, *p* = 0.2), the descriptive statistics indicate the current pattern of the data originates mostly from the doctor with an accurate self-perception being perceived more self-deprecatory compared to the poet with a balanced view of self. These results imply that, for an expert source, disclosing a minor limitation, even when presented in combination with merits, could raise the perception of self-deprecation relatively easily.

Third, the impact of accuracy induction for perceived conventionality was much larger when the source was a doctor [*F*(1, 247) = 6.9, *p* < 0.001, η^2^ = 0.03; *M* = 3.22, *SD* = 0.69, *n* = 130 for the accuracy, and *M* = 2.71, *SD* = 0.81, *n* = 129 for the inaccuracy condition] rather than a poet [*F*(1, 280) = 1.24, *ns*; *M* = 3, *SD* = 0.69, *n* = 143 for the accuracy, and *M* = 2.78, *SD* = 0.76, *n* = 148 for the inaccuracy condition]. These results imply that lowering the self in a consistent manner can be accepted unconventional – at least within the sub-population of the American culture – especially when the source was a doctor with expertise that matters.

Combining the three observations, it could be inferred that unconditionally discounting one’s competence may *not* be a wise impression management strategy for an expert source as it could dampen the intention to interact, raise the perception of self-deprecation more than intended, and be conceived as unconventional. Specifically, noting that perceived conventionality of the speech is inversely related to perceived self-deprecation (*r*_*doctor*_ = 0.27, *p* < 0.001) but positively associated with all the major outcome measures (see Table 5), it could be further inferred that self-deprecating can also lower the attraction of the source as such a tactic is considered unconventional for an expert source like a doctor in the U.S.

## Discussion

The present study sought to provide an alternative way of establishing the construct validity of humility particularly by demarcating it from self-deprecation or unconditional self-underrating. Humility was conceptualized as a latent construct subsuming accurate self-assessment, open-mindedness, and egalitarianism. The analyses compared perceived humility to perceived self-deprecation on selected criterion measures. The current study makes an important contribution to the theory and research on humility through its design. Specifically, the study attempted to construct-validate humility by employing participant responses to a self-introduction written to represent true humility instead of relying on self-report measures to reduce biases.

The current findings should assist scholars to examine humility in a more precise manner and more importantly as a *communication* construct rather than one based on self-assessment (see Kruse et al., 2017). In the main, results indicate that humility differs from self-deprecation in that humility induces the perception of sincerity more strongly than self-deprecation; self-deprecation suppresses the perception of narcissism more powerfully than humility; perception of humility associates positively with self-consciousness while the perception of self-deprecation relates negatively with self-consciousness. Humility behaved differently from self-deprecation when correlated with measures of source credibility, interpersonal attraction, and the intention to interact with the source. Perceived humility tended to enhance all three attributes of source credibility (i.e., perceived expertise, trustworthiness, and goodwill) and interpersonal attraction (i.e., task, social, physical), and the intention to interact to a similar extent, whereas perceived self-deprecation lowered perceived expertise, task attraction, and the intention to interact, and produced minimal impact on the personality dimensions of source credibility (i.e., trustworthiness, goodwill) and social/physical attraction.^2^

## Limitations and Future Studies

Current findings were somewhat inconsistent with the negative view of self-deprecation. Unlike the prediction, participants did *not* necessarily find self-deprecation insincere, narcissistic, or self-conscious. This inconsistency seems attributable to a sample characteristic; sophomores may evaluate speech less critically than would, say, communication professionals specialized in politeness or pragmatics, or individuals with complex cognition, for whom inferring unstated premises or motivations from what is said is a major part of their job (e.g., a political analyst) or intellectual fun (e.g., the armchair politician). Future replication studies employing such a critical audience might be able to document this. Measuring and controlling for participants’ level of criticality or cognitive complexity could also be helpful.

That students with Western cultural origin comprised the majority of the current sample could also explain the inconsistency. Self-enhancement is more normative in Western cultures, while self-criticizing, an intensive form of self-deprecating, is more common in East Asia (e.g., Kitayama et al., 1997). To the extent that self-deprecating is normative *and* normal, most East Asians should exercise self-deprecation to conform to the norm and expect the same from others. That self-deprecation is enacted due to the external pressure translates that self-underrating is unlikely to affect the receiver’s view of the source (i.e., they expect it from them); the source is simply observing the norm and yet hides the true judgment of self. The belief that people behave in a humble manner due to normative pressures could result in a substantial depreciation of self-underrating among East Asians. Findings from a replication study involving an East Asian sample should determine the validity of this conjecture.

The gender of the speaker was left ambiguous instead of being subjected to systematic manipulation. Given participant assessments of source credibility, source attractiveness, and perceived sincerity might vary based on the gender identity of the speaker (D’Errico, 2020; D’Errico et al., 2022), we encourage future research to investigate potential gender differences. Also, noting the multi-dimensionality of humility, there may exist other important components constituting the construct (e.g., low focus on the self and high focus on others, Liu, 2016; Wright et al., 2017). Such additional dimensions, however, were not fully explored in the current study for the simplicity of the research design. Future scholars are recommended to identify and incorporate them into the experiment to see how they behave when correlated with other major components of humility. As well demonstrated in more recent research, elements of humility can be found outside the linguistic domain, including non-verbal communication cues, such as facial expression, body posture, and emotion (see D’Errico and Poggi, 2019; Scardigno et al., 2021). Finally, the current investigation solicited participation from college students primarily raised in the United States. A future study could include a non-student sample from different cultures to determine the generality of how humility is perceived.

## Conclusion

The current study sought to provide practical advice for sources inquiring about an efficient self-presentation strategy. Humility should receive merited attention for the potential to resolve Blau’s (1960) dilemma: *‘How can a person attract, or remain interpersonally approachable, to an audience impressed with their expertise?’* Past findings recommended that expert sources lower the barrier by adopting self-deprecatory tactics. Findings from the present study, however, provide counterevidence that self-deprecation could result in a lowered interpersonal attraction. Moderate self-deprecation might enhance approachability as evidenced in the previous literature, but the current data indicate that this outcome might have resulted because self-underrating hampered perceived expertise – hence the source portrayed as less skilled than necessary – rather than increasing their trustworthiness or perceived caring. That is, self-deprecation cues might help attract people as claimed but *at the expense of* perceived competence. A source might lose little for self-deprecating if their level of expertise is insignificant to their audience, for example, strangers on an airplane who believe they will never meet again. However, for those whose expertise can readily affect their audience (e.g., a doctor or an attorney), self-deprecating might lead to being perceived as less competent and in turn substantially less credible.

Humility cues, on the other hand, seem to enhance a source’s interpersonal attractiveness by raising *both* their perceived expertise and trustworthiness, although the magnitude in which they mediate the impact might vary depending on how persuasive the source’s expertise is for the receiver. The effect could be more pronounced for interactants who are of unequal power (e.g., teacher-student or doctor-patient relationships) or where the relationship between parties is of great importance for achieving goals (e.g., members of a team). Regardless, the source’s expertise will likely be perceived as having future value to the receiver. Recent research offers support for the idea that perceived humility in experts, such as doctors, can have positive health implications. For example, clinician humility during medical encounters might lead to increased patient satisfaction, trust, and subjective assessment of health conditions (see Huynh and Dicke-Bohmann, 2020).

## Data Availability Statement

The raw data supporting the conclusions of this article will be made available by the authors, without undue reservation.

## Ethics Statement

The studies involving human participants were reviewed and approved by University of Wisconsin-Milwaukee IRB. The patients/participants provided their written informed consent to participate in this study.

## Author Contributions

S-YK developed the original research idea and collected and analyzed the data. ES conducted a literature search and review. Both authors agreed to be accountable for the content of the work.

## Conflict of Interest

The authors declare that the research was conducted in the absence of any commercial or financial relationships that could be construed as a potential conflict of interest.

## Publisher’s Note

All claims expressed in this article are solely those of the authors and do not necessarily represent those of their affiliated organizations, or those of the publisher, the editors and the reviewers. Any product that may be evaluated in this article, or claim that may be made by its manufacturer, is not guaranteed or endorsed by the publisher.

## Footnotes

1 All significance tests involving product-moment correlations were conducted after Fisher’s *z*-transformation.

2 Humility impact for physical attraction was relatively small and significantly positive only for the doctor (*r* = 0.18). The impacts of self-deprecation on interpersonal attraction were qualified by the source occupation; it tended to lower the task attraction for the doctor (*r* = -0.32) and raise the social attraction for the poet albeit weakly (*r* = 0.12).
