# Supplementary material for: Construct-Validating Humility: Perceptions of a Humble Doctor
Source: Front Psychol. 2022 May 17;13:882622. doi: 10.3389/fpsyg.2022.882622 (PMC9152537; doi:10.3389/fpsyg.2022.882622)
Supplement: Supplementary file 1 [file Data_Sheet_1.PDF]

Author	Year	E/C	Accurate/Balanced Self-Assessment	Open-Mindedness	Egalitarianism	Remarks
Baumeister & Exline	2002	C			Permanent shrinking of the self. Self-loss.	
Ben-Ze'ev	1993	C	Not overestimates his/her worth. Knows his/her superior worth. Does not deny one's superior position or merits. Does not hide accomplishments.	Tolerant of beliefs and practices of other people which differ from one's own.	Evaluates his/her fundamental human worth as similar to that of other people. Egalitarian. Evaluate one's limitations as comparable with those of others.	Modesty used to refer to humility. Demarcates humility from self-deprecating. Humble persons are sincere and less concerned about how they are viewed by others.
Casey	2001	C	No need lies and evasions to inflate one's importance in the eyes of their associates. Equally content with both the gifts and the limitations that come from their nature.	Have overcome the tendency to regard others as competitors. Work with whatever they have. Waste no time envying those who possess different qualities.	"The term 'humility' is related to the word 'humus' and points to a connectedness with the earth and, by extension, with all that inhabits the earthly sphere" (p.1).	
Driver	1989	C	A humble person, unlike a modest person, can paint an accurate, though perhaps unflattering, picture of himself. The man who is unduly humble [modest] falls short of an accurate appraisal of himself.			
Emmons & Kneezel	2005	C				Discussed humility in relation with gratefulness and surrender to the creator or a power greater than oneself.

Author	Year	E/C	Accurate/Balanced Self-Assessment	Open-Mindedness	Egalitarianism	Remarks
Emmons	1999	C	Realistic appraisal of one's strengths and weaknesses, neither overestimating nor underestimating them.<br>Accurate opinion of oneself.<br>Not to have a low opinion of oneself.<br>To have a sense of self-acceptance and an understanding of one's imperfections.		Ability to keep one's talents and accomplishments in perspective.	
Emmons	2007	C			A recognition that we could not be who we are or where we are in life without the contributions of others.	Discussed humility in relation with gratefulness and surrender to the creator or a power greater than oneself.
Fullam	2009	C	Not the act of self-abasement or minimizing one's own capacities or achievements.<br>A prudential mean between two contrary vicious extremes i.e., contextless self-abnegation versus excessive self-focus.	Humility entails a kind of truth-claim about our self-assessment, specifically of our own deficiency.	Self-knowledge acquired by turning one's attention outside oneself.<br>We learn who we are by other-centeredness.<br>Humility invites us to be awake and aware of the skills, gifts, and the virtues of others, especially when we do not possess them ourselves.	Refutes the attempt at connecting humility to religiosity stating.<br>Puts self-knowledge at the center or the beginning point from which one can end up acquiring true humility.<br>*Humility is a relational or interpersonal virtue as constant comparison leads to an accurate self-assessment.*

Author	Year	E/C	Accurate/Balanced Self-Assessment	Open-Mindedness	Egalitarianism	Remarks
Gantt	1967	C	Not contradictory to self-confidence and independence of thinking.	Open mind toward the opinions and facts by others.<br>A capacity for self-criticism and a lack of proprietary feeling towards one's pet theories and concepts.<br>Humble scientists subject their concepts to critical evaluations through free discussion.<br>"I am of those who seek knowledge and are willing to learn." *Peter the Great.*	"I feel like a little child playing by the seashore while the great ocean of truth lies undiscovered before me." *Isaac Newton.*	Humility discussed in practice of healthy science.
Hare	1996	C	Accurate assessment of one's own relative moral superiority.<br>A quality of making accurate self-assessments, often with special emphasis on non-overestimation, as opposed to underestimation, of one's merits.			Discussed moral humility
Harrell & Bond	2006	C	Sensibility with respect to the breadth and depth of our knowledge.<br>Acknowledging one's limitations.<br>Willingness to identify limitations and to experience of feelings of vulnerability.	Acknowledging what we do not know frees us to be open to new and an anticipated learning.<br>Readiness to connect and learn.<br>Willingness to take critical perceptions seriously.	Deep respect for the community.<br>Understanding of the multiple contextual forces.	Discussed 'empowered' humility in relation with community engagement.
Means, Wilson, Sturm, Biron, & Bach	1990	C	Recognition that one cannot control every situation.<br>Not a decrease in the valuation of oneself.	Willingness to admit one's real inadequacies.<br>Patience and gentleness with others.	Genuine empathy considering how one's actions affect others.<br>Increase in the valuation of others.	Humility discussed as a means of psychological treatment for the interpersonally maladaptive.

Author	Year	E/C	Accurate/Balanced Self-Assessment	Open-Mindedness	Egalitarianism	Remarks
Morgan	2001	C			Lack of self-overestimation must be supplemented with (a) some principle of fundamental human equality and (b) the overall collective value of human beings in the larger scheme of things or the assumption that human value is comparatively low from Christian or a religious standpoint.	Humility is NOT a secular virtue, which is thinkable only in reference to the transcendent or God.
Morris, Brotheridge, & Urbanski	2005	C	Crest of human excellence between arrogance and lowliness.' Ability to understand one's strengths and weaknesses.	Willingness to learn from others.	Acceptance of something greater than the self. Understanding of the small role that one plays in a vast universe, an appreciation of others, and a recognition that others have a positive worth.	
Murray	2001	C			"…it is simply the sense of entire nothingness that comes when we see who truly God is everything." (p. 17)	"Humility is not the same as low self-esteem and it is not the opposite of confidence. In fact, the truly humble person walks with absolute confidence, knowing that we are simply empty vessels through whom God wants to accomplish his work. When we understand true humility, we understand that it is not about us at all. It is about God." (p. 7).

Author	Year	E/C	Accurate/Balanced Self-Assessment	Open-Mindedness	Egalitarianism	Remarks
Murray	2005	C			The sense of entire nothingness. "I am nothing."	Humility as entire dependence on god, first duty and the highest virtue of the creature and the root of every virtue, and the nature of God. Provides no set definition of humility.
Neuringer	1991	C	Humility does not require self-abasement or lowly demeanor nor prohibit assertion. Overstatements and overgeneralizations by Thorndike, Watson, Pavlov, and Skinner are partly to blame for the rejection of behavioral work by non-behavioral psychologists.	Should communicate with scientists of other fields and rely on intersubjectivity (Two-Labs strategy).		Characterizes humble behavioral scientists. Humble descriptions of procedures and results more persuasive.
Peterson & Seligman	2004	C	Accurate, not underestimated, sense of one's abilities and achievements. Ability to acknowledge one's imperfections and limitations. Not involves self-disparagement.	Openness to new ideas, contradictory information, and advice	Keeping one's abilities and accomplishments in perspective. Relatively low focus on the self. Ability to 'forget the self.' Appreciation of the value of all things, as well as the many different ways that people and things can contribute to our world.	

Author	Year	E/C	Accurate/Balanced Self-Assessment	Open-Mindedness	Egalitarianism	Remarks
Powers, Nam, Rowatt, & Hill	2007	E	Accurate assessment of one's own characteristics.	Ability to acknowledge limitations.<br>Relinquishes one's own arrogant or narcissistic tendencies.<br>Acknowledges mistakes.	Forgetting of the self.<br>Views oneself realistically in relation to others.<br>Respects others.<br>Relates to spiritual transcendence, which refers to the ability to stand outside of their immediate sense of time and place to view life from a larger, more objective perspective.	Humility discussed in relation with forgiveness and spirituality.
Richards	1992	C	"Humility too involves having a accurate sense of oneself, sufficiently firm to resist pressures toward incorrect revisions." (p.5)<br>"…there are difficulties with analyzing humility as having a low opinion of oneself, and that we would think of it instead as an inclination to keep one's accomplishments, traits, and so on in unexaggerated perspective, even if stimulated to exaggerate." (p. 8)	A humble person with accurate yet realistic self-assessment will not suffer with jealousy or envy when surpassed by other superior individuals for he/she not presumes that he/she should be able to compete on equal terms. (p. 194)<br>"No doubt it[humility] should open a person to the possibility that he does not know the best, that his information is incomplete or his inferences faulty." (p. 198)	"A person who is a good judge of his or her own achievements and virtues should also be a good judge of others [without feeling pressures to either over- or undervalue the deeds of others as well] (p. 17)"<br>"Humility also provides an understanding that one is not special...not an exception to be treated differently from others."	Demarcates humility from 'false humility' (self-deprecation), which tends to lead into an overdo of the pretense (e.g., lowering the self excessively and unnecessarily) because the pretender should act to make the audience to believe so.<br>Considers humility as a virtue praising the honesty and courage to maintain and state their accurate judgment of their self-achievement in front of overrated adulations.
Roberts	1982	C			Humility involves "the disposition to gladly construe as my equal every person who is presented to me." (p. 67)	

Author	Year	E/C	Accurate/Balanced Self-Assessment	Open-Mindedness	Egalitarianism	Remarks
Rowatt, Ottenbreit, Nesselroade Jr., & Cunningham	2002	E	Realistic appraisal of one's positive and negative characteristics.		Measured humility examining the magnitude of self-other bias (e.g., overvaluing the self in relation to others or considerably undervaluing others in relation to the self).	Humility discussed in relation with religiousness.
Rowatt, Powers, Targhetta, Comer, Kennedy, & Labouff	2006	E		Intellectually open.<br>Willingness to admit imperfections.<br>Open-minded.<br>Openness to different perspectives or scientific discoveries.	Respectful of others.<br>Lack of self-focus or self-serving biases.<br>Less egotistical.	
Sandage	1999	C	"A realistic orientation toward self and other, that includes a willingness to acknowledge one's strengths and face one's limitations." (p. 262)		"Ego-humility is a non-defensive, self-differentiated orientation that facilitates courageous engagement of relational dialogue." (p. 262)	Defined EGO-humility

Author	Year	E/C	Accurate/Balanced Self-Assessment	Open-Mindedness	Egalitarianism	Remarks
Sandage & Wiens	2001	E	A realistic self-orientation that includes a willingness to acknowledge one's strengths. Should be differentiated from false humility of perpetual self-denigration or a need for self-abasement or self-disparagement.	Face one's limitations.	Views others as one's equal. The differing gifts within the body of Christ with each member's gifts being important to the overall body. Unselfishness.	Humility discussed in relation with Paul. Philippians 2:3: *Humility is not to be confused with false modesty, or with that kind of abject servility that only repulses...Rather, it has to do with a proper estimation of oneself...Here one is well aware both of one's weaknesses and of one's glory (we are in his image, after all) but makes neither too much nor too little of either. True humility is therefore not self-focused at all, but rather, defined by Paul in v.4, "looks not to one's own concerns but to those of others." (p. 188)*
Sedikides, Gregg, & Hart	2007	C	Moderate view of self neither too positive (i.e., self-enhancement) nor too negative (i.e., self-effacement)			Used the term modesty to mean humility.
Tangney	2000	C	Accurate self-assessment of one's abilities. Ability to acknowledge one's limitations.	Openness to new ideas.	Seeing oneself as just one person in the larger scheme of things. Appreciation of the values of other individuals. Lack of self-focus.	

Author	Year	E/C	Accurate/Balanced Self-Assessment	Open-Mindedness	Egalitarianism	Remarks
Templeton	1995	C	Not self-deprecation.<br>Knowing you were created with special talents and abilities to share with the world.	Our ability to admit we can never know everything or be all things to all people.<br>Must be rooted in the knowledge and understanding of the limits of our personal power.<br>Open to learn from others and to refuse to see issues and people only in blacks and whites.	You are one of many, each with an important part to play in life.<br>Accepting you have personal power but are not omnipotent.	
Vera & Rodriguez	2004	C	Acknowledges one's limitations.<br>Not repels adulation.	Not narcissistic.<br>Eager to learn from others.<br>Asks for advice.<br>Open to new paradigms.	Respect others.	
von Hildebrand	1976	C	"…we are nothing by ourselves…everything we have is received [from God]…still we have received a great deal from God." (p. 41).<br>Grow aware of one's importance.	"Dependence on other men by no means evokes in him a sense of oppression…Nor does it embarrass him to have to ask someone's pardon or to confess a wrong he has done." (pp. 84-85)	"He knows that he has received whatever good there is in him from God, and attributes nothing to himself." (p. 69).<br>Does not feel in any way superior to others.	Acknowledges humans' creaturely status and dependence on god.

E/C: E = Empirical; C = Conceptual
